# Neurologists’ views on patient reported outcomes in multiple sclerosis care

**DOI:** 10.1016/j.heliyon.2022.e09637

**Published:** 2022-06-02

**Authors:** Signe Baattrup Reitzel, Marie Lynning, Lasse Skovgaard

**Affiliations:** The Danish Multiple Sclerosis Society, Poul Bundgaards Vej 1. st., DK-2500, Valby, Denmark

**Keywords:** Patient reported outcomes, Multiple sclerosis, Complex disease, Patient involvement

## Abstract

**Background:**

The value that patient reported outcomes (PROs) can bring to the clinical encounter is increasingly being recognized. Within the field of Multiple Sclerosis (MS), a number of activities have been initiated internationally with the aim of integrating PROs in MS care. Integration of PROs in MS care will, among other things, require MS neurologists' acceptance of PROs. This qualitative study aimed to explore MS specialized neurologists’ view on the potentials and barriers for the use of PROs in the clinical setting.

**Methods:**

Eight neurologists specialized in MS participated in a series of individual in-depth semi-structured interviews. Interviews were audio-recorded and transcribed. A thematic analysis was conducted using a reflexive thematic approach to identify meaning units and themes emerging from the interviews. The analysis focused on barriers and potentials for PROs that relate to the specific characteristics of MS.

**Results:**

Three main themes emerged from the data. “The role of PROs in describing the patient's situation” describes how informants perceived MS as a complex disease, and in this context, PROs can bring forth new or otherwise hidden information, but they may also blur the picture. The theme “The validity of PROs reported by MS patients may be questionable” describes how impairments caused by MS, such as cognitive and physical disabilities, were identified as risk factors that could cause unreliable self-reported outcome measures within this patient group. Finally, the theme “Involving the patient” regards how the clinical conversation was viewed as the most important source of patient reported information, but at the same time PROs carry the potential to enhance shared decision making.

**Conclusion:**

This study indicates that, according to MS neurologists, integration of PROs in MS clinical practice, though possibly valuable, is not without challenges. Possible benefits of PROs include the ability to bring forth otherwise hidden information on the patient's health status and the enhancement of shared decision making. Barriers include difficulties in capturing the full situation of the patient via PROs due to the complexity of MS as well as the issue of various MS-related impairments compromising the validity of PROs reported by MS patients.

## Highlights

• The complexity of MS affects neurologists' attitudes towards PROs.
• PROs hold the potential to bring forth otherwise hidden information.
• Neurologists are sceptical regarding the quality of information from PROs.
• PROs may not provide sufficiently detailed information about patients' situations.

## 1 Introduction

Patient reported outcomes (PROs) are aimed at systematically capturing patients' perspectives [1]. PROs are information on the patient's health, quality of life, or functional status associated with health care or treatment directly reported by the patient without interpretation of the patient's response by a clinician or anyone else [2]. Including patients' perspectives ensures a more holistic assessment of health status. PROs have been recognized as a valuable tool alongside biomarkers and may be used as a supportive communication tool for health professionals in understanding patients' experiences [2, 3].

Multiple sclerosis (MS) is characterized by a multitude of symptoms and conditions that affect patients' wellbeing. Objective measures of disease burden traditionally used in MS care, such as the Expanded Disability Status Scale (EDSS), do not always reflect patients’ experience of their health [4], and patients with MS believe that PROs hold important potentials, such as enhanced patient involvement and more multifaceted agendas in clinical consultations [5].

Integration of PROs in health services has received increasing interest from policymakers in recent years [6]. In a Danish context, health authorities have initiated standardization of the clinical use of PROs in the health care system. This work will, in time, cover all disease areas, including MS. The Danish standardization of PROs in clinical practice was initiated in 2015 with the project “Program PRO” [7].

However, the wish to capture patients’ perspectives through PROs faces various challenges [1]. Disease progression and cognitive decline might challenge patient reports [8], and for a disease like MS, characterized by large variability in impairments, relevant PROs can be difficult to identify and develop [1].

Clinicians' acceptance of PROs is paramount if the potentials of PROs in clinical care are to be realized, and PROs must be tailored to the specific needs of both clinicians and patients to ensure their successful implementation [9, 10, 11]. Clinicians' attitudes toward the use of PROs in the clinic have been examined in the fields of cancer, palliative care and mental health and to some extent within acute care, rheumatoid arthritis and Parkinson's disease [12, 13, 14]. Most studies identify divergent attitudes among clinicians towards PROs. Concerns include increased workload, integration of PRO(s) into existing practices, and the potential negative impact of PROs on the patient-clinician relationship. In terms of advantages, clinicians believed that PROs can improve communication, help identify relevant information and create structure and prioritization in the consultation [12, 14]. It seems that the existing literature about health professionals' attitudes toward PROs touch upon themes that go across several disease areas. Within MS, only one study has included clinicians' attitudes toward PROs. This study used survey data to evaluate the benefits of the PRO tool ‘MSdialog’, an electronic PRO Diary in MS [4]. However, attitudes towards PROs in general and not a specific PRO tool have, to our knowledge, not been studied in neurologists treating MS, and an assessment of PROs with a focus on specific characteristics of MS is lacking.

Within the MS field, PROs are currently used in many different forms in several tools, including but not limited to MSIS-29, LMSQoL, MSWS-12, FAMS, HAQUAMS, MUSIQoL, PRIMUS and MSQoL [15, 16]. An international initiative, Patient Reported Outcomes for MS (PROMS), has been established to reach international consensus on how, and by which tools PROs can be integrated into clinical practice as well as regulatory agencies’ decision making processes [17].

This study aims to inform the ongoing work to implement PROs in MS care with insights into MS neurologists’ views on potentials for and barriers to the use of PROs in MS care, with a focus on aspects related to the specific characteristics of MS.

## 2 Materials and methods

This study was a qualitative interview study performed among Danish neurologists specialized in MS.

### 2.1 Recruitment strategy

The head of the Danish MS registry was asked to nominate relevant candidates for the study. Informants were recruited via e-mail invitations using a purposive sampling strategy. From the list of nominated neurologists’ relevant candidates were invited to participate in the study. The relevance of candidates was decided based on an attempt to ensure a geographical spread in order to represent regional differences in MS care and a variation in prior experiences with PROs [18]. Invitations were sent to the relevant neurologists until a satisfying sample, representing all Danish regions and differing PRO experiences, was reached. This resulted in the inclusion of eight neurologists, including the head of the Danish MS registry.

### 2.2 Data collection

In-depth interviews were carried out in the spring of 2019. The interviews were semi-structured, allowing for a detailed exploration of the neurologists' perspectives on PROs. An interview guide was developed with the aim to seek insight into the neurologists' attitudes towards PROs for MS care. The interview guide was developed as collaboration between the authors and Associate professor Henriette Langstrup. The interview guide was structured as general themes to discuss with the informants and possible questions to inform the dialogue. The themes covered in the interview guide included: clinical practice in MS care, value-set, experience and thoughts on PROs, data quality, and needs. The term ‘PRO’ was not defined or explained to the neurologists unless prompted as we wished to capture the informants' perspectives on PROs without drawing attention to specific tools or purposes. Eight interviews were undertaken. The length of the interviews varied between 35 and 80 min.

### 2.3 Data analysis

All interviews were audio-recorded and transcribed verbatim. However, unfinished sentences with no meaningful content and expressions such as “hmm” and “uhm” were left out.

The interviews revolved around informants’ views on PROs for clinical use in broad terms. However, as a delimitation we chose to focus this analysis on perceived barriers and facilitators as they relate to the specific characteristics of MS according to the informants. Thus, statements regarding more general concerns, such as the added burden of registration and data management or the integration of PRO data into existing work procedures, were not included in the analysis.

Themes were extracted using a reflexive thematic approach as suggested by Braun and Clarke [19]. The reflexive thematic approach consists of six phases. Firstly, the transcriptions were thoroughly read through in order to get a sense of the of the whole. Fragments of the transcripts were then qualitatively coded using an inductive approach to clarify meaning units expressed by the informants (phase two). In phase three, the codes from the second phase were categorized into draft themes. These themes were revised and changed in light of new insights (phase four) until we defined the final suitable themes in phase five. In the sixth phase, the results of the analysis were reported [15]. The thematic analysis was undertaken in an iterative process, and phases were continuously revisited, and themes were discussed among the authors until consensus was reached [18].

To ensure validity and reliability of the results, independent analyses were performed by authors SBR and ML and discussed until consensus was reached [18]. The analysis was conducted using QSR NVivo 12 software.

Quotes reported in this paper are translated from Danish into English by the authors using a contextualized hermeneutic translation technique to ensure that the translations accurately capture the meaning and experience of the participants.

### 2.4 Ethics, consent, and permissions

Informed, written consent to participate in the study was obtained from all study participants. Participation was voluntary. Participants were assured of confidentiality and anonymity and were free to withdraw from the study at any time. The study adhered to the EU General Data Protection Regulations.

## 3 Results

Informants from all five administrative regions, who run the public hospitals and thereby the 14 MS clinics in Denmark, participated. Five out of the eight participants were female. Participant characteristics are presented in Table 1. Most of the informants did not have prior experience with systematic use of PROs, although several had used ad hoc patient questionnaires as preparation for clinical consultations. One informant (Informant 7) had previously used PROs as part of data collection for a PhD research project, and one informant (Informant 2) had previous experience with PROs as a tool to select patients for control visits.

**Table 1:** Participant characteristics.

	Gender	Age	Region of practice	Previous PRO experience
Informant 1	Female	47	North Denmark Region	No prior use of PROs but use patient questionnaires in consultations.
Informant 2	Male	51	Central Denmark Region	No prior use of PROs but use patient questionnaires in consultations.
Informant 3	Female	63	Capital Region of Denmark	No prior use of PROs but use patient questionnaires in consultations.
Informant 4	Male	50	Region of Southern Denmark	No prior use of PROs.
Informant 5	Female	56	Region Zealand	No prior use of PROs but use patient questionnaires in consultations.
Informant 6	Female	50	Capital Region of Denmark	No prior use of PROs. It is a field of great interest.
Informant 7	Male	56	Region Zealand	From research.
Informant 8	Female	42	Region of Southern Denmark	No prior use of PROs but use patient questionnaires in consultations.

Three themes emerged from the analysis, providing an overall picture of MS neurologists’ perspectives on PROs used in MS clinical care (Table 2).

**Table 2:** Themes and sub-themes.

Themes	Sub-themes
The role of PROs in describing the patient's situation	PROs can bring forth new information
	PROs may blur the picture
The validity of PROs reported by MS patients may be questionable	Disabilities as a barrier against reliable reporting of PROs
	Compromised self-assessment
	Enhancing the validity of PROs
Involving the patient	The clinical conversation is PRO
	PROs as a tool for shared decision making

### 3.1 Theme 1: the role of PROs in describing the patient's situation

The informants described MS as a complex disease because it manifests in numerous physical and cognitive symptoms and disabilities.

The first theme illuminates how the complexity of MS as a disease leads to an ambivalence in the informants' perception of whether and how PROs can enhance the understanding of MS patients' situations. On the one hand, they considered PROs to be a tool to obtain a more nuanced understanding. On the other hand, they expressed doubts regarding PROs’ ability to capture the most important information from the patients.

#### 3.1.1 PROs can bring forth new information

Half of the informants indicated that they saw in PROs a potential to add a new dimension of patient provided information to the clinical conversation. According to these informants, PROs can help create a more complete picture of the patient's state of health over time than what can be elicited in the clinical conversation alone.

*“I see it as a possibility to obtain more elaborate information in some areas that aren’t covered within a short conversation. […] When we meet the patients, we only get a snapshot. If you get consecutive reports [using PROs] from the patients, you can get a more detailed understanding of their disease or how it varies over time.”* (Informant 8)

In addition, a few informants mentioned that PRO tools may help structure the clinical conversation so that important information is not lost or forgotten.

*“It [having filled in a questionnaire before the consultation] can be helpful in the sense that it creates a structure and gives the patient the opportunity to prepare for the consultation, making sure that the relevant topics are covered.”* (Informant 4)

*“Self-report – that would be fantastic. The patients know their own body best, and they may not always be so well articulated when they see the doctor. Some patients have a lot of anxiety or phobias and aren’t able to articulate themselves [in the situation]”* (Informant 5)

#### 3.1.2 PROs may blur the picture

In contrast, a few informants stressed that PROs will not be able to provide a sufficiently detailed picture of a complex disease such as MS.

*“MS is a disease that can result in all sorts of neurological symptoms, in numerous combinations and degrees of severity […] It is a disease with thousands of faces”* (Informant 2)

They emphasized that an attempt to use PROs to summarize a patient's situation will result in inadequate information. Alternatively, it would require PRO questionnaires that are very comprehensive.

*“I question whether a questionnaire can exist that comprises pivotal questions that will be important to everyone. And how will you interpret it later when you do not know the factors that influence the answers that have been given? […] I need to create a relation to the patient so I can get an understanding of the patient’s experiences […] And I have to translate that into neurology-language so I can get a sense of whether it has anything to do with MS. […] [In a conversation] I can easily figure out what is meant by sensory disturbances. But if I have to do it via a questionnaire, I would have to give around 10 options for what these sensory disturbances could be and then it would become very comprehensive.”* (Informant 7)

It was further emphasized that there is a surprisingly big difference between MS patients. Their life and situation are often complex, and the informants stressed that this cannot be captured in a questionnaire.

*“I still think we need a doctor who meets the patients where they are. Each patient is different. They are in different phases of their life and may have different wishes for their lives and their course of illness. It’s an extremely complex situation that cannot always be uncovered by a questionnaire.”* (Informant 4)

### 3.2 Theme 2: the validity of PROs reported by MS patients may be questionable

The informants considered MS to be a disease which, due to its complex ways of affecting the brain and the body, could compromise the information gathered from patients via PRO tools. According to the informants, many MS patients experience functional and cognitive impairments and a lack of insight into their own state of health, which could compromise their ability to report information that reflects their true situation.

#### 3.2.1 Disabilities as a barrier against reliable reporting of PROs

Most of the informants expressed concern that cognitive impairments, fatigue, and physical disabilities may affect MS patients’ ability to complete PRO questionnaires and provide valuable and valid information.

Since MS is a disease that affects the brain, this may have implications for the ability of patients to express how the disease affects them. One informant describes it this way:

*“Perhaps MS is so complex, compared with other diseases, because it takes place in the brain – the same brain that has to think about the symptoms and explain them* […] *We have some patients whose cognitive abilities are affected to such an extent that they simply can’t fill in or understand such things* [PRO tools]*”* (Informant 1)

In addition, it was argued that PROs might overwhelm some patients who might not have the energy to answer a long questionnaire and that excess fatigue constitute a barrier again sufficient reporting of PROs.

*“Yet another challenge for some of our patients is that they do not have the energy to answer a bunch of questionnaires. […] With these patients I have to adapt my questions to their capabilities.”* (Informant 2)

#### 3.2.2 Compromised self-assessment

A few informants pointed out that as MS manifests in the brain it might affect the patients’ ability to fully comprehend the effects of the disease on their health situation. Two examples of this is self-assessed walking ability and quality of life assessments as expressed by informants 4 and 7:

*“We have this EDSS score, but the walking function is often assessed based on an estimate by asking the patient how far they can walk [as a substitute for an actual walking test]. A group in Aarhus has conducted a systematic research project investigating the reliability of these estimations and found that they [patients] quite often get it wrong.”* (Informant 4)

*“There is always a huge amount of adaptation to new life circumstances once people get sick. Then the new situation becomes the norm. […] we know that from questions about quality of life.”* (Informant 7)

#### 3.2.3 Enhancing the validity of PROs

Some informants suggested different strategies to improve PRO reports. For example, it was suggested that PROs could be reported by next of kin or a caregiver. Alternatively, the patients could be educated to ensure that they possess the sufficient competencies to assess disease activity.

*“If they have certain disabilities, it could be that a nurse or a relative could fill in the questionnaire with the patient. […] If patients have not received the right tools or information on how to use it [the PRO tool], we will get doubtful information that we cannot use. The patients must receive tools to assess when they experience a worsening in their disease”* (Informant 5)

### 3.3 Theme 3: involving the patient

The informants agreed that perspectives from MS patients should play a central role when deciding upon a treatment strategy. The clinical conversation was seen as the most important source of information from the patient, and some of the informants viewed the conversation itself as a type of PRO. At the same time, some informants viewed PROs as information to supplement the clinical conversation, and they indicated that it could contribute to enhancing shared decision making.

#### 3.3.1 The clinical conversation is PRO

Due to, among other things, the complexity of MS, all informants considered it a core task to engage patients in the clinical encounter and ask patients to contribute excessively throughout the clinical conversation. The informants considered this conversation to be the most important form of information exchange with their patients.

*“Neurology is a specialty where it is absolutely crucial that we talk to our patients and gain information from them. It is essential for our ability to assess how things are. […] so, there is a lot of conversation. That’s where I spend most of the time.”* (Informant 2)

*“It [the clinical conversation] is the most important patient reported outcome. I believe I can read a lot between the lines and from their body language and the ways they react to my questions. There is a lot of information that isn’t verbal.”* (Informant 4)

Some informants equated patient provided information obtained in the clinical conversation with PROs in the sense that the conversation entails a structured and standardized collection of relevant information from the patient.

*“[…] they [patients] can contribute with whatever is on their mind […]. When that is over, I have a template in the back of my mind, and then I systematically ask questions […] because that is part of the data I have to register.”* (Informant 7)

#### 3.3.2 PROs as a tool for shared decision making

The informants indicated that the treatment of MS is complex as there are many different treatment options to consider, which affects how the neurologists guide the patients in the clinical conversation.

*“Now we have fourteen very different treatment options – pills, injections, infusions, given with different frequencies, with different effects, and with different side effects of course.”* (Informant 7)

According to the informants, patients should play an active role in deciding their treatment. They explained how the neurologist takes the lead and presents the options they think is best for the specific patient while being open to the preferences of the patient, especially when it comes to the possible adverse effects of a treatment.

*“The patient is taken seriously and receives information about the treatment options. It is a joint decision, but of course I will give professional guidance. But they also get the chance to consider other options”* (Informant 5)

A few informants indicated that PROs may be a tool to improve shared decision-making because it can inform clinicians about aspects they would otherwise not be aware of or because PROs, on an aggregate level, can inform patients about the experiences of other patients using a certain drug.

*“…we cannot avoid the fact that the patient’s perception of the treatment plays a role in what treatments they will accept. Here PROs may help us gain insight into some side effects or circumstances of life that are not otherwise revealed. […] PROs will not be deciding whether the patient should shift to this or that treatment, but it could be a tool to include the perception of the patient”* (Informant 8)

*“…if we systematically collect quality of life data for each treatment type, perhaps it can help us to conclude that “most people are doing well on this treatment”. That would be a helpful tool to tell the patients how other patients experience the treatment.”* (Informant 8)

## 4 Discussion

This qualitative study illuminates neurologists' views on opportunities and barriers, relating to the specific characteristics of MS, for the use of PROs in clinical care. The results point to an ambivalence regarding the feasibility and appropriateness of PROs for MS. On the one hand, PROs may bring forth nuances and important information about the patients that would otherwise be lost. Thus, PROs could strengthen treatment decisions and be used as a tool to improve shared decision making. On the other hand, PROs may not be adequate in capturing the most important aspects of the patients' situation. Also, due to the disease burden, MS patients’ ability to report valid health information may be compromised.

### 4.1 Potentials and barriers

The results of this study indicate that MS neurologists ascribe significant importance to the patient-physician communication taking place during a consultation. In that connection, they refer to PROs as a possible tool to provide additional information from the individual patient's perspective. Greiner et al. found similar indications in their study, emphasizing the potential embedded in the PRO tool ‘MSdialog’ to improve the communication and sharing of information between the MS patient and the MS health care professionals [4]. Damman et al. investigated patients' and clinicians' attitudes toward using PROs in clinical care for Parkinson's disease [13] – a neurological disease which carries some similarities to MS. They found that clinicians saw potential in using PROs to monitor individual changes over time which would allow patients better insight into their own disease progression, improve monitoring of treatment effects, and support personalized care and shared decision making [13]. The informants in the present study similarly emphasized that PROs hold the potential to bring forth otherwise hidden information to the clinical encounter, and in agreement with Damman et al., it was indicated that PROs may be used as a tool to improve shared decision making.

Although the neurologists see a potential in the application of PROs to MS care, they are at the same time sceptical regarding the quality of the information brought forth by PROs. Their scepticism relates to the multifaceted and complex characteristics of MS, which make it difficult for PRO tools to capture all relevant aspects of the patient's situation. These barriers are similar to those described by D'Amico et al., who recognize that the variable nature of MS necessitate a wide spectrum of PROs in order to obtain a full understanding of patients' situation [1]. For PROs to capture all aspects of MS, the tools would have to be very substantial. An alternative to a comprehensive and unmanageable PRO tool is the development of shorter PRO tools focusing only on certain domains with particular importance for neurologists and patients. The neurologists in this study underlined that MS patients' ability to report self-assessed health information may be compromised due to patients' impairments, slow disease progression and MS′ impact on the patients' brain. Others have also pointed to the potential issues associated with the use of self-reported outcomes in patients with cognitive impairments [8, 20]. These concerns suggest that clinical use of PROs in the field of MS requires careful consideration of patients' cognitive status.

Part of the solution to some of the above issues could lie in the use of digital tools which allow for easy, in-the-moment registration, such as ecological momentary assessments [21] or passive data collection with activity and sleep trackers [22]. Westergaard et al. have shown that the use of digital tools among PwMS allows for frequent patient-reported data to be collected effectively, but that the overall registration burden imposed on participants when taking this approach should be carefully considered [23].

This study reveals that MS neurologists perceive significant barriers to successful use of PROs in MS care which points to the importance of involving neurologists in all aspects of planning and implementation of PROs, as well as ensuring a high level of transparency regarding the purpose of implementing PROs in MS care. This was also emphasized by Boyce et al. in a systematic review of health professionals’ experience with PROs, including studies on both physicians and nurses treating patients within various disease areas other than MS [12]. Boyce et al. further underline that, from a health care professional perspective, practical barriers to the routine use of PROMs may occur when the correct infrastructure is not in place before commencing data collection or when their use is disruptive to normal work routines [12]. In Denmark, the MS area is relatively small and well organized and to elude a situation where PROs disrupt work routines neurologists with responsibility for workflow planning should be involved in the processes around PRO development and implementation.

Based on a qualitative study among PwMS, Westergaard et al. emphasize the importance of combining patients' and health care professionals' perspectives regarding PRO if aiming at sustainable solutions [5]. In the present study, several neurologists articulate the importance of patient involvement, and it should be considered that such involvement also includes patients’ active participation in the preparation and implementation of PROs.

### 4.2 Strengths and limitations

A strength of this study was the purposive sampling strategy which is an acknowledged recruitment strategy for qualitative studies [18]. Purposive sampling allowed for a diverse informant group in regard to gender, age, location, and previous experience with PROs. This strategy, despite the small study population, ensured varied perspectives on PROs in MS care. A sample including more participants with thorough experience with PROs could have been advantageous. The study only included one participant with clinical experience in systematic use of PROs in MS care. This reflects upon the fact that systematic inclusion of PROs is still very limited in Danish MS care. However, several of the informants were familiar with some sort of PRO as either a research tool or “dialogue starter” in clinical encounters. Further, the reliability and validity of the analysis was enhanced by the involvement of multiple researchers [18].

Some of the neurologists may have been wary of disclosing views that conflicted with norms. It was mentioned by several informants that PROs constitute a trending and politically correct phenomenon – a perception which may have led them to express less criticism towards the usefulness of PROs. However, both positive and negative attitudes towards PROs were obtained in the interviews, which suggests that this was not a major limitation.

### 4.3 Implications

The challenges highlighted in this study emphasize the need for efforts to identify and/or develop appropriate PRO measures for use in clinical MS care that are both suitable for patients with MS while also being viewed by clinicians as useful in the clinical encounter. For this work, initiatives such as PROMS, which bring together patients, neurologists and other relevant actors in developing and planning PROs, are of essential value [17].

To align patients' and clinicians’ expectations towards PROs, clear information and instructions are needed to allow for greater transparency regarding the purpose of PROs.

## 5 Conclusion

According to Danish MS neurologists, the integration of PROs in MS clinical practice can be a valuable tool by bringing forward new information regarding patients' health status and enhancing shared decision making. Though found valuable, integration of PROs will, according to neurologists, not come without challenges. Particularly in a complex disease like MS, the neurologists fear that it will be difficult to capture the full situation of the patient through PROs. For PROs to be successful, strategies should be put in place to ensure that neurologists with thorough knowledge about MS patients’ abilities are sufficiently included in planning and implementation of PROs to be used in MS care.

## Declarations

### Author contribution statement

Signe Baattrup Reitzel: Conceived and designed the experiments; Performed the experiments; Wrote the paper.

Lasse Skovgaard, Marie Lynning: Conceived and designed the experiments; Analyzed and interpreted the data; Wrote the paper.

### Funding statement

This work was supported by the 10.13039/100008361Danish MS Society.

### Data availability statement

Data will be made available on request.

### Declaration of interests statement

The authors declare no conflict of interest.

### Additional information

No additional information is available for this paper.

## Figures and Tables

**Table:** 

Temaer	Forskningsspørgsmål	Probing spørgsmål
Baggrunds-oplysninger	Kan du starte med at fortælle mig lidt om dig selv og dit job.	• Hvor ofte ser du MS-patienter • Hvor længe har du arbejdet på MS-afdelingen • Har du ledelsesansvar i din nuværende stilling • Arbejder du både klinisk og med forskning • Hvad er din alder
Praksis	Kan du beskrive en typisk konsultation Hvordan indgår data i dit arbejde Hvordan er arbejdsdelingen på afdelingen	• Hvor lang tid har du pr. patient - Hvor meget tid er med patienten, hvor meget er forberedelse inden • Hvordan forbereder du dig på en konsultation • Hvilke patient”typer” møder du i dit arbejde • Hvilke data/mål/prøver anvender du • Hvad bruger du data til • Hvor meget fylder dataarbejde foran skærmen • Deles data om patienters helbred - fx med fysioterapeuter eller i forhold til best practice/kvalitetssikring • Er der et hierarki forbundet med arbejderopgaverne
Værdier	Hvilke værdier guider dig i dit arbejde	• Hvad er vigtigst for dig i dit arbejde med MS-patienter • Er der andet der også er vigtigt/spiller en rolle for dit arbejde • Hvordan bør forholdet mellem læge og patient se ud
PRO-data	Hvad ved du om PRO Har du erfaring med PRO Hvilke fordele/ulemper er det ved PRO (det du tænker er PRO.) – evt fordele ulemper ved anden PRO anvendelse Hvilken værdi kan PRO give Hvad forestiller du dig PRO vil betyde for nuværende arbejdsgang Er der nogen patienter, der ikke kan bidrage med PRO data Hvilke ændringer vil det kræve at implementere PRO som del af behandlingen af MS-patienter Hvad er din holdning til at anvende PRO som visitationsværktøj?	• Hvad tænker du formålet med PRO er • Hvordan tror du PRO fungerer • Hvilke oplysninger • I hvilken forbindelse • Stiller du spørgsmål til dine patienter i konsultationen • Hvad kan PRO tilføre dit arbejde • Hvad ser du som værende PROs styrker • Hvilke faktorer er en barrierer for at anvende PRO i behandlingen af MS-patienter • Hvad tænker du om at PRO kan give indsigt i forskellige forhold du ikke kan løse nu og her – hvordan er dine muligheder for at føle du hjælpe patienten videre • Er der et specifikt problem du tænker PRO kan være med til at løse • Vil PRO medfører ændringer i dine opgaver • Vil PRO medfører ændringer i arbejdsdelingen på afdelingen • Kan alle patienter anvende PRO (hvorfor ikke) • Hvilke ændringer vil det kræve af dig i dit arbejde • Hvilke ændringer vil det kræve af afdelingen • Hvilke ændringer vil det kræve af sundhedssystemet • F.eks. patienten udfylder spørgeskema og rangeres efter grøn, gul, rød hvilket bestemmer hvorvidt de skal ind til kontrol (AMBUFLEX)
Datakvalitet	Hvad karakteriserer data af høj kvalitet Kan patienter leverer valid data	• Hvilken data er mest relevante for dig/i dit arbejde • Er der noget data, der er mere anvendeligt end andet • Har subjektive data værdi/data fra patienten • Hvor meget må patienter influere på data
Behov	Er der noget relevant viden du mangler i dit arbejde Hvordan skal PRO værktøjet se ud for at være meningsfuldt i din hverdag	• Er der noget specielt du har brug for at vide om dine patienter • Hvordan skal data præsenteres hvis du skal anvende det i dit arbejde (diagrammer, farvekoder ect.) • Hvordan skal det indhentes (før/i konsultation/kontrol) • Med hvilken frekvens skal data indsamles

**Table:** 

Theme	Research questions	Probing questions
Background information	Can you start by telling me a bit about yourself and your job?	• How often do you see MS patients? • How long have you worked in the MS department? • Do you have managerial responsibility in your current position? • Do you work both clinical and with research? • How old are you?
Practice	Can you describe a typical consultation? How is data included in your work? How is the division of labor in the department?	• How long do you have per. patient – How much time do you spend with the patient – how much time do you use on preparation? • How do you prepare for a consultation – which patient “types” do you meet in your work? • What data/measures/samples do you use? • What do you use data for? • How much of your time is spent in front of the screen? • Are patients' health data shared – e.g., with physiotherapist or in relation to best practice/quality assurance • Is there a hierarchy associated with the work tasks
Values	Which values guide you in your work?	• What is the most important for you, in your work with MS-patients? • Is there anything else that is important or plays a role in your work? • How should the relationship between doctor and patient look like
PRO-data	What do you know about PRO? Do you have any experience with PRO? What are the advantages/disadvantage of PRO (what you think PRO is) – e.g., advantages/disadvantages of other PRO use What value can PRO provide What do you imagine PRO will mean for the current work procedure? Are there any patients who cannot contribute PRO data? What changes would it require to implement PRO as part of the treatment of MS patients What is your opinion on using PRO as a visitation tool?	• What do you think the purpose of PRO is? • How do you think PRO works? • Which information • In which connection • Do you ask your patients questions during the consultation? • What can PRO bring to your work • What are PROs strengths • Which factors are a barrier for the use of PRO in the treatment of MS patients? • What do you think about gaining insights from PRO about issues you can't help with right now – what are your options for directing your patient to another health care professional? • Is there a specific problem you think that PRO cannot solve? • Will PRO cause any changes in your tasks? • Will PRO entail any change in division of labor in the department? • Can all patients use PRO (why not)? • What changes will it require of you in your work • What changes will it require in the department • What changes will it require of the health system • For example. The patients fill in the questionnaire and is ranked according to green, yellow, and red witch determines whether they should go for a check-up (AMBUFLEX)
Data quality	What characterizes high quality data Can patients provide valid data	• Which data is most relevant to you/in your work? • Is there any data that is more useful than anything else? • Has subjective data value/data from the patient • How much can the patients influence data
Need	Is there any lack of relevant knowledge in your work? What should the PRO tool look like to be meaningful in your everyday life	• Is there anything special you need to know about your patients? • How should data be presented if you are to use it in your work (diagrams, color codes etc.) • How to obtain it (before/in consultation/control) • At what frequency should data be collected
